# Gold Nanoparticle-Based Colorimetric and Fluorescent Dual-Mode Lateral Flow Immunoassay for SARS-CoV-2 Detection

**DOI:** 10.3390/jfb15030058

**Published:** 2024-02-27

**Authors:** Ying Gan, Hefan Zhang, Jing Liu, Fuqin He, Fengheng Li, Ao Li, Man Xing, Dongming Zhou, Shan-Yu Fung, Hong Yang

**Affiliations:** 1 The Province and Ministry Co-Sponsored Collaborative Innovation Center for Medical Epigenetics, Tianjin Key Laboratory of Inflammatory Biology, Department of Pharmacology, School of Basic Medical Sciences, Tianjin Medical University, No. 22 Qixiangtai Road, Heping District, Tianjin 300070, China; 2 State Key Laboratory of Experimental Hematology, Key Laboratory of Immune Microenvironment and Disease (Ministry of Education), Department of Immunology, School of Basic Medical Sciences, Tianjin Medical University, No. 22 Qixiangtai Road, Heping District, Tianjin 300070, China; 3 Biosensor National Special Laboratory, Key Laboratory for Biomedical Engineering of Ministry of Education, Department of Biomedical Engineering, Zhejiang University, Hangzhou 310027, China; 4 Department of Pathogen Biology, School of Basic Medical Sciences, Tianjin Medical University, Tianjin 300070, China

**Keywords:** lateral flow immunoassay, gold nanoparticles, dual-mode analysis, RNA detection, SARS-CoV-2

## Abstract

Severe acute respiratory syndrome coronavirus 2 (SARS-CoV-2) infection caused the COVID-19 pandemic, impacting the global economy and medical system due to its fast spread and extremely high infectivity. Efficient control of the spread of the disease relies on a fast, accurate, and convenient detection system for the early screening of the infected population. Although reverse transcription–quantitative polymerase chain reaction (RT-qPCR) is the gold-standard method for SARS-CoV-2 RNA analysis, it has complex experimental procedures and relies on expensive instruments and professional operators. In this work, we proposed a simple, direct, amplification-free lateral flow immunoassay (LFIA) with dual-mode detection of SARS-CoV-2 RNA via direct visualization as well as fluorescence detection. The viral RNA was detected by the designed DNA probes to specifically hybridize with the conserved open reading frame 1ab (ORF1ab), envelope protein (E), and nucleocapsid (N) regions of the SARS-CoV-2 genome to form DNA–RNA hybrids. These hybrids were then recognized by the dual-mode gold nanoparticles (DMNPs) to produce two different readout signals. The fluorescence characteristics of different sizes of GNPs were explored. Under the optimized conditions, the LFIA presented a linear detection range of 10^4^–10^6^ TU/mL with a limit of detection (LOD) of 0.76, 1.83, and 2.58 × 10^4^ TU/mL for lentiviral particles carrying SARS-CoV-2 ORF1ab, E, and N motifs, respectively, in the fluorescent mode, which was up to 10 times more sensitive than the colorimetric mode. Furthermore, the LFIA exhibited excellent specificity to SARS-CoV-2 in comparison with other respiratory viruses. It could be used to detect SARS-CoV-2 in saliva samples. The developed LFIA represents a promising and convenient point-of-care method for dual-mode, rapid detection of SARS-CoV-2, especially in the periods with high infectivity.

## 1. Introduction

Coronavirus disease 2019 (COVID-19) caused by severe acute respiratory syndrome coronavirus 2 (SARS-CoV-2) infection [1] has been a huge challenge for the global healthcare system and has impacted on the economy as well as many social problems. SARS-CoV-2 is an RNA virus in the Betacoronavirus genus of the family Coronaviridae [2,3]. It contains a single RNA genome encoding four structural proteins for the spike (S), envelope (E), membrane (M), and nucleocapsid (N) [4]. This virus has unique properties of high transmissibility, strong infectivity, and a relatively long incubation period, which facilitate the fast and wide spread of the virus [5,6]. Although the COVID-19 pandemic is currently under control, to prevent the potential next outbreak of SARS-CoV-2 and its mutants, an effective way to detect this viral infection accurately, cost-effectively, and conveniently is still necessary.

Reverse transcription–quantitative polymerase chain reaction (RT-qPCR) is known as a gold-standard method to detect SARS-CoV-2 with high sensitivity and specificity [7,8]. However, such a method has complex procedures consisting of RNA extraction, primer design, RNA reverse transcription to cDNA, and real-time DNA amplification. It also relies on expensive instruments and experienced technicians for operation [9,10]. Moreover, it usually takes several hours to days to obtain the results from the collected samples, which is a critical factor for the early control of the virus spread. Thus, there is still a need for a cost-effective, simple, and point-of-care testing (POCT) method to detect SARS-CoV-2 for the disease control.

The lateral flow immunoassay (LFIA) represents one ideal methodology for POCT as this assay is fast, low-cost, and user-friendly [11,12]. In the past decades, the LFIA has been widely used in various fields, including SARS-CoV-2 detection [13,14,15,16]. For example, Wu’s group reported a signal amplification system using catalytic hairpin assembly (CHA) reaction coupled with an LFIA-based fluorescence detection method to detect SARS-CoV-2 in oropharyngeal swab samples with a limit of detection (LOD) of 2000 viral RNA copies/mL [14]. Yan’s group designed a microfluidic device coupled with commercial pregnancy test strips to analyze SARS-CoV-2 RNA as low as 500 copies/mL with the help of reverse transcription–loop-mediated isothermal amplification (RT-LAMP) [13]. Although these LFIAs were able to detect SARS-CoV-2 with high sensitivity, the signal amplification reactions used in these methods were complicated and time-consuming. Recently, Wang’s group proposed a simple and rapid LFIA for SARS-CoV-2 detection using an anti-DNA–RNA hybrid monoclonal antibody (S9.6) possessing high affinity to DNA–RNA duplexes without any amplification steps [16]. This assay could achieve extremely high sensitivity and specificity for both throat swabs and sputum samples with a LOD of 1000 TU/mL. However, this method only allows for the fluorescent mode of detection, which requires an instrument for the readouts.

To make the test strips more reliable and convenient to use, dual-mode detection of LFIA has been proposed for SARS-CoV-2 detection [17,18,19,20,21,22]. For instance, Xiao’s group proposed a colorimetric and fluorescent dual-function LFIA for S protein detection with a novel dual-functional immune label fabricated by coating a single-layer shell formed by mixing gold nanoparticles (GNPs) and quantum dots on a SiO_2_ core [19]. Wang’s group developed a colorimetric–fluorescent dual-mode LFIA for the rapid, sensitive, and simultaneous detection of SARS-CoV-2-specific IgM and IgG in human serum using S protein-conjugated SiO_2_@Au@QD nanobeads [21]. While these studies set good examples for the improvement of the LFIA’s performance by incorporating multiple detection signals in one device, they were designed and developed for the detection of SARS-CoV-2 antigens or antibodies. Studies have shown that the sensitivity of antigen detection is relatively low [23,24], and antibody testing is prone to false-negative results at the early stage of infection due to individual differences in immune responses [25,26,27]. In contrast, SARS-CoV-2 RNA detection possesses the advantages of high accuracy and sensitivity [28], and the development of a dual-mode LFIA that allows for both colorimetric and fluorescence detection of SARS-CoV-2 RNA is anticipated to benefit current COVID-19 diagnosis as well as the control of the virus spread.

In this work, we developed a simple, direct, amplification-free colorimetric and fluorescent dual-mode LFIA for SARS-CoV-2 RNA detection based on functionalized dual-mode gold nanoparticles (DMNPs). In our system, DNA probes were used to capture the SARS-CoV-2 RNA to form DNA–RNA hybrids, which were identified by the S9.6 antibody (which is more efficient than another anti-DNA–RNA hybrid antibody D5H6 [29,30]) immobilized on the test line of a strip. The DMNPs were made of the GNP cores modified with the fluorescent Cy5 probes and the S9.6 antibodies, which were able to bind with the DNA–RNA hybrids captured on the test line. Once the DMNPs were accumulated on the test line of the strip, they could be visualized as a red color band or a fluorescent band (on a fluorescence imager) for the dual-mode detection of SARS-CoV-2 RNA. For the proof of principle, three different SARS-CoV-2 lentiviral particles that carried the E, N, and the open reading frame 1ab (ORF1ab) regions of the SARS-CoV-2 RNA genome were fabricated. Under the optimized conditions, these lentiviral particles were detected with an LOD of about 10^4^ TU/mL in the fluorescent mode, which was up to 10 times more sensitive than that in the colorimetric mode. Furthermore, our device showed excellent specificity in distinguishing SARS-CoV-2 from other respiratory viruses. We also demonstrated that the developed LFIA was able to analyze SARS-CoV-2 in saliva samples. This work developed a novel, convenient, and efficient LFIA method for SARS-CoV-2 detection, which allows for self-testing at home with the naked eye and more sensitive fluorescence quantification for clinical uses, especially in the periods of high infectivity.

## 2. Experiments and Methods

### 2.1. Chemicals and Reagents

All reagents used in this work were analytical-grade unless otherwise noted. Gold (III) chloride trihydrate (HAuCl_4_·3H_2_O), sodium borohydride (NaBH_4_), and a polyethylenimine (PEI) transfection reagent were purchased from Sigma-Aldrich (St. Louis, MO, USA). Bovine serum albumin (BSA), Tween-20, tris(hydroxymethyl)aminomethane, sodium chloride (NaCl), magnesium chloride (MgCl_2_), goat anti-rabbit IgG, and rabbit IgG (H + L) were bought from Sangon Biotech (Shanghai, China). Phosphate-buffered saline (PBS), tris-hydrochloride buffer, Triton X-100, and polybrene were purchased from Solarbio (Beijing, China). Sodium citrate tribasic dihydrate (C_6_H_5_O_7_Na_3_·2H_2_O) was purchased from BBI (Shanghai, China). The S9.6 antibody was purchased from Kerafast (Shirley, MA, USA). Cy5-PEG5000-SH was obtained from Fanshuo (Harbin, China). The lateral flow strip was purchased from Taiyu (Shanghai, China). The peptide of P12 (CLPFFD) used to stabilize the GNPs was synthesized by Jietai (Nanjing, China) [31]. Dulbecco’s Modified Eagle Medium (DMEM) and fetal bovine serum (FBS) were purchased from Biological Industries (Beit Haemek, Israel). 

The sequences (5′-3′) of the DNA probes referring to Wang’s work [16] were synthesized by GenScript (Nanjing, China) (Table 1).

Three plasmids carrying the target DNA sequences (pCDH-E154, pCDH-N283, and pCDH-ORF1ab), the envelope plasmid pMD2.G, and the packaging plasmid psPAX2 used for the production of lentiviral particles were all provided by HonorGene (Changsha, China). The target sequences (5′-3′) of pCDH-E154, pCDH-N283, and pCDH-ORF1ab [16] are listed in Table 2.

### 2.2. Synthesis of DMNPs and the Control Nanoprobes

GNPs with diameters of 5 nm (GNP5), 13 nm (GNP13), and 20 nm (GNP20) were synthesized following the method reported previously in [32]. For GNP5’s synthesis, a HAuCl_4_ solution (25.4 mM; 972 μL) and trisodium citrate (34 mM; 2 mL) were added to 90 mL of ultrapure water. NaBH_4_ (19.8 mM; 1 mL) prepared with an ice-cold trisodium citrate solution (34 mM) was added to the mixture and stirred for 10 min (800 rpm; 0 °C). The mixture was kept undisturbed overnight at room temperature before use. For GNP13’s synthesis, a HAuCl_4_ solution (1.066 M; 161.2 μL) was added to 200 mL of ultrapure water. The mixture was heated to boiling under stirring (800 rpm), followed by the addition of trisodium citrate (340 mM; 2 mL). After 15 min, the solution was allowed to cool to room temperature. For GNP20’s synthesis, a HAuCl_4_ solution (1.066 M; 23.8 μL) was added to the 100 mL of ultrapure water and heated to boiling. Trisodium citrate (34 mM; 5 mL) was added to the mixture. After 30 min, the solution was cooled to room temperature. The concentration of the synthesized GNPs was determined by a micro-spectrophotometer (Allsheng, Hangzhou, China).

For the fluorescence characterization, 3.2 μL, 10.1 μL, and 11.8 μL of BSA (1%) was first added to GNP5 (85 nM; 500 μL), GNP13 (10 nM; 2 mL), and GNP20 (1.4 nM; 7 mL) to stable the nanoparticles, respectively, in which the amount of BSA per unit area of each size of GNP was the same. After 4 h, 1.80 μL, 0.85 μL, and 0.41 μL of Cy5 (1 mM) was added to GNP5, GNP13, and GNP20, respectively, in which the ratio of the number of GNPs to the added Cy5 was equal to 45:1. At the same time, 7.5 μL, 23.8 μL, and 27.6 μL of P12 (1 mM), and 2 μL, 6.3 μL, and 7.36 μL of BSA (1%), was added to the corresponding GNPs. After 24 h, the GNPs were blocked with 10% BSA followed by centrifugation, twice.

To fabricate the DMNPs, the pH of the GNP5 solution was adjusted to 8 with a K_2_CO_3_ solution (0.1 M). The S9.6 antibody (4 μL; 8 mg/mL) was added to the GNP5 solution (85 nM; 500 μL), followed by the addition of BSA (1%; 2 μL), P12 (7.5 μL; 1 mM), and Cy5-PEG5000-SH (1 mM; 1.8 μL). All the Cy5 used in this work was conjugated with PEG5000 to increase the distance between the GNPs and Cy5. The mixture was incubated in the dark for 24 h. To block the unbounded sites, 10% BSA was added again to the GNP5. After 1 h, the DMNPs were purified by centrifugation (14,000 rpm), twice. Similarly, the control nanoprobe was prepared by mixing the rabbit IgG (4.7 mg/mL, 8 μL) with the pH-adjusted GNP5 (500 μL). After 24 h, the control nanoprobe was obtained by centrifugation at 14,000 rpm, twice. The collected DMNPs and the control nanoprobes were redispersed in 10 mM of PBS (1% BSA) and then centrifuged and filtered to remove large particles. 

The sizes and morphologies of the GNPs and DMNPs were characterized by dynamic light scattering (DLS) analysis (Zetasizer, Malvern Instruments, Worcestershire, UK) and transmission electron microscopy (TEM, JEOL, Tokyo, Japan), with an accelerating voltage of 80 kV.

### 2.3. Production of Lentiviral Particles Carrying Three Different SARS-CoV-2 RNA Sequences

HEK293T cells were cultured in DMEM supplemented with 10% FBS at 37 °C and 5% CO_2_. The cells were seeded into 10 cm culture dishes at a density of 6 × 10^6^ cells/dish the day before transfection. The packaging plasmids psPAX2 and pMD2.G and the expression plasmids pCDH-ORF1ab, pCDH-E154, and pCDH-N283 were thoroughly mixed with the PEI transfection reagent. The mixtures were added to the cells and incubated at 37 °C and 5% CO_2_ for 6 h. The culture medium (containing the produced lentiviral particles carrying the SARS-CoV-2 RNA sequences of interest) was collected at 60 h after transfection and was further filtered (0.45 μm) and concentrated by ultrahigh-speed centrifugation (27,000 rpm). The lentiviral particles were re-suspended in ice-cold PBS and stored at −80 °C for future use.

### 2.4. Determination of the Transduction Titer of Lentiviral Particles 

HEK-293T cells were seeded in a 6-well plate at a density of 6 × 10^5^ cells/well and cultured overnight. On the following day, the cells in each well were transduced with packaged lentiviral particles with five different dilutions (20, 200, 2000, 2 × 10^4^, and 2 × 10^5^). Polybrene with a concentration of 8 μg/mL was added to the wells. After 6 h, the transduction media were replaced with fresh DMEM with 10% FBS. After 48 h, the cells were scraped into centrifuge tubes, centrifuged, and rinsed, followed by re-suspension with PBS containing 2% FBS. The proportion of green fluorescent protein (GFP)-positive cells was detected in each group with a flow cytometer (LSRFortessa, BD, San Jose, CA, USA) and analyzed with the FlowJo v10.8.1 (TreeStar, Ashland, OR, USA) software. The transduction titer of the lentiviral particles was calculated according to the following formula:

$$Virus\;Titer\left(TU/mL\right)=(cell\;transduced\times \%positive\times dilution\;factor)/100$$

### 2.5. The LFIA’s Fabrication

Based on the card-based assembly that was prepared by the Taiyu company, the S9.6 antibody and goat anti-rabbit IgG (2 mg/mL) were dispensed on a bare card as the test line and the control line, respectively, using a platform dispenser (XYZ3035, Jinbiao, Shanghai, China). The antibody solution was dried and cured on the card at 37 °C for 2 h. The assembled card was cut into individual strips with a width of 3 mm. The strips were kept dry at 4 °C before use.

### 2.6. Dual-Mode Detection of Lentiviral Particles Carrying Three Different SARS-CoV-2 RNA Sequences

Lentiviral particles (5 μL) were mixed with the detection buffer, which contained the DNA probes (2 μL; 10 μM), Triton X-100 (15 μL; 2%), MgCl_2_ (2.2 μL; 0.2 M), Tris-HCl (0.3 μL; 1 M), and NaCl (6 μL; 5M). The mixture was incubated at 56 °C and then cooled to room temperature for lentiviral lysis and RNA hybridization. Next, the mixture containing the DNA–RNA hybrids (30 μL) was added to the sample pad of the strip. After 10 min, PBST (20 μL; 1% Tween-20) was added to rinse the strip, followed by the addition of DMNPs and control nanoprobes (12 μL). The detection results were visualized by taking a picture for colorimetric analysis and using the ChemiDoc Imaging System (Bio-Rad, Hercules, CA, USA) for fluorescence analysis. Image J 1.51j8 was utilized to obtain the B value of the test line based on RGB splitting for the colorimetric analysis. Image Lab 3.0 was used to measure the average fluorescence intensity of the band for the fluorescence analysis.

### 2.7. Statistical Analysis

GraphPad Prism 7.0 was used for statistical analysis. All data were expressed as means ± SEM, and *p* < 0.05 was considered statistically significant. For two-group comparisons, Student’s t-test was performed, while one-way ANOVA with the Bonferroni post-test was used for multiple comparisons. 

## 3. Results and Discussion

### 3.1. The Design and Workflow of LFIA

**Scheme 1:**
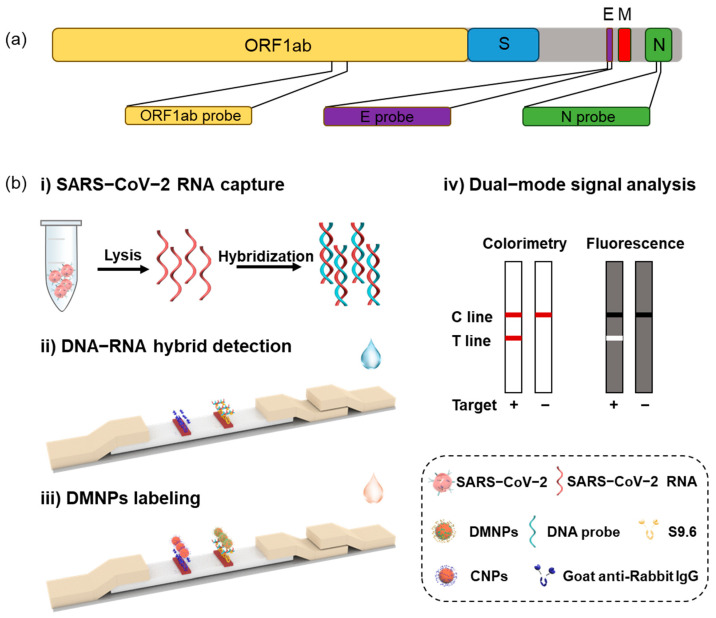
The schematic workflow of the proposed dual-mode LFIA for the detection of SARS-CoV-2. (**a**) Genome map showing the ORF1ab, E, and N DNA probes that hybridize with the three gene regions (ORF1ab, E, and N) in the SARS-CoV-2 RNA genome. (**b**) The workflow of LFIA. SARS-CoV-2 RNA is released and bound with DNA probes forming DNA–RNA hybrids, which can be captured by S9.6 antibodies on the test line of the strip. Upon the DMNPs binding, the colorimetric and fluorescent signals can be obtained.

Our study aimed to develop a convenient and simple LFIA sensing platform for SARS-CoV-2 detection with high sensitivity and specificity. Three DNA probes (ORF1ab probe, E probe, and N probe) that specifically target the ORF1ab, E, and N regions of the SARS-CoV-2 RNA genome were designed to detect SARS-CoV-2 RNA to improve the detection accuracy (Scheme 1a) [33]. In this work, lentiviral particles containing the sequences of these three target genes served as pseudo-typed SARS-CoV-2. In the presence of SARS-CoV-2 lentiviral particles, the DNA probes bound to the corresponding gene region and formed the DNA–RNA hybrids after SARS-CoV-2 RNA was released. The LFIA was pre-coated with the S9.6 antibody and goat anti-rabbit IgG antibody on the test line and the control line of the strip, respectively. The control nanoprobes were made of GNPs modified with rabbit IgG bound to the control line to determine the validity of the strips. On the test line, when the DNA–RNA hybrids in a sample flowed through the strip, they were captured by the S9.6 antibody and then further probed by the DMNPs, which were made of GNPs functionalized with the S9.6 antibody and fluorescent Cy5-PEG5000-SH. Enabled by the dual-mode (colorimetric and fluorescent) of the DMNPs, the LFIA was able to measure the target genes by both the naked eye and a fluorescence device, allowing for the POCT method’s application in various scenarios and providing a useful tool for the prevention of the spread of infection (Scheme 1b). 

### 3.2. Validation of the Performance of the Synthesized DMNPs in RNA Detection

**Figure 1:**
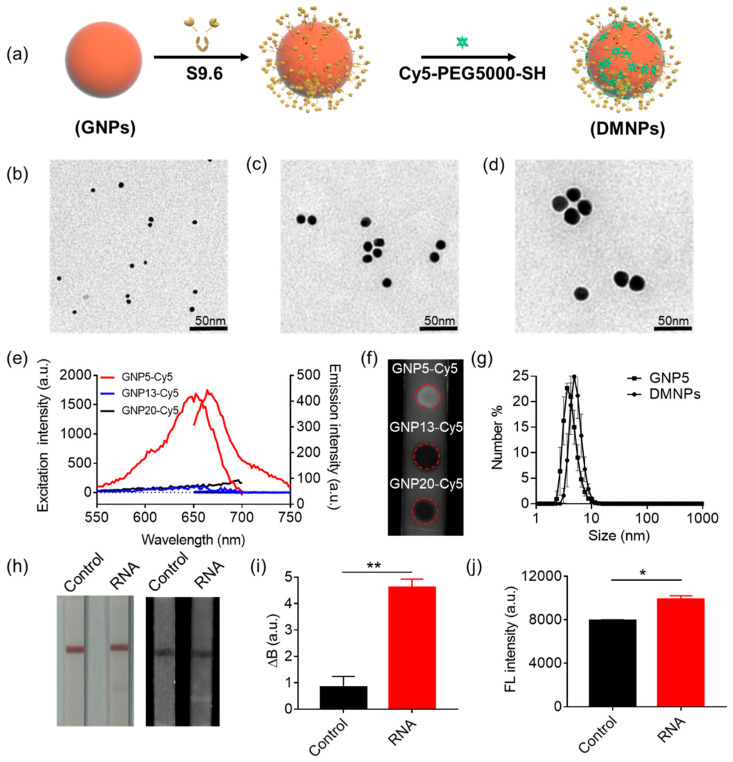
The physicochemical characterization of DMNPs and the evaluation of their performance in RNA detection. (**a**) A schematic diagram of DMNPs’ synthesis. The TEM images of GNP5 (**b**), GNP13 (**c**), and GNP20 (**d**). (**e**) The fluorescence intensity scan of three sizes of GNPs with Cy5. Excitation scan wavelength: 550–700 nm (emission wavelength: 750 nm). Emission scan wavelength: 650–800 nm (excitation wavelength: 600 nm). (**f**) Fluorescence images of dried droplets of GNP5–Cy5, GNP13–Cy5, and GNP20–Cy5 on the nitrocellulose membrane. (**g**) The hydrodynamic diameter of GNPs and DMNPs by DLS measurement. Scale bar: 50 nm. (**h**) Bright-field (left) and fluorescent (right) images of DMNP-based LFIA for direct RNA detection. (**i**) Colorimetric and (**j**) fluorescence analysis of (**h**). ΔB = |B_background_ − B_test line_|. N ≥ 3 per group; * *p* < 0.05; ** *p* < 0.01.

DMNPs were fabricated with GNPs modified with the S9.6 antibody and Cy5-PEG5000-SH, where the GNPs allow for direct colorimetric visualization by the naked eye and Cy5 allows for fluorescence detection (Figure 1a). GNPs of a size of 13 nm or larger are usually utilized in LFIAs to generate optical signals [34,35]. However, this size may not be suitable for the preparation of fluorescent gold nanoparticles due to the possible quenching effect. To choose an optimal size, three sizes of GNPs (5 nm, 13 nm, and 20 nm) were synthesized. As shown in the TEM images (Figure 1b–d), the diameters of the GNPs were measured to be 4.80 nm ± 1.19 nm, 12.49 nm ± 1.69 nm, and 22.01 nm ± 2.29 nm, respectively, confirming the successful synthesis of the three sizes of GNPs. Next, we compared the fluorescence characteristics of the three sizes with BSA as the model protein (mimic S9.6 antibody), in which the amount of BSA per unit area of each size of GNP remained consistent, and the number ratio of the added GNPs to Cy5 was 45:1 (the same as for the DMNPs). It can be seen that GNP5–Cy5 exhibited a strong fluorescence intensity at the excitation (646 nm) and emission (664 nm) wavelengths, while almost no fluorescence was observed in GNP13–Cy5 or GNP20–Cy5 at the same concentration (50 nM) (Figure 1e). It was reported that when fluorophores were placed near metal surfaces, resonant energy transfer took place, resulting in the acceleration of the radiative decay rate [36]. Furthermore, larger GNPs usually possess a stronger quenching capability due to the increased overlap of the dye’s emission spectrum with the surface plasmon resonance of the GNPs [37,38]. Thus, GNP13 and GNP20 may quench more Cy5 compared with GNP5 and lead to a low fluorescence emission even though the PEG5000 on the Cy5 is used to increase the distance between the GNPs and the Cy5. To further verify the quenching phenomenon, a droplet of GNPs–Cy5 was placed on the strip and dried under 37 °C. The fluorescent image shows that only GNP5–Cy5 generated a bright fluorescent spot, while the other sizes of GNPs quenched Cy5 with a black spot (Figure 1f), which is consistent with the fluorescence intensity scan. 

Based on the excellent fluorescence characteristics of GNP5–Cy5, DMNPs were finally synthesized using GNP5. As shown in Figure 1g, the hydrodynamic size and dispersibility were characterized by DLS. Upon the modification of the GNPs with the S9.6 antibody and Cy5, the hydrodynamic size increased from 7.66 nm ± 0.19 nm to 12.31 nm ± 0.73 nm with polydispersity indices (PDIs) of 0.10 ± 0.05 and 0.46 ± 0.03, respectively, indicating the good stability of the prepared DMNPs. Next, based on the verification of the fluorescence characteristics, the performance of the DMNPs in RNA detection in the LFIA was evaluated. Compared with the control group, the DMNPs were able to bind to the DNA–RNA hybrids captured on the test line of the LFIA and exhibited a red band as well as a bright fluorescent band (Figure 1h). The results of the colorimetric (ΔB_RNA_/ΔB_control_ = 5.5) and fluorescence intensity (FL_RNA_/FL_control_ = 1.2) analyses show that the signal values with the addition of the RNA (RNA group) were both significantly higher than that of the control group (Figure 1i,j), confirming the capability of the DMNPs to report the presence of a specific RNA.

### 3.3. SARS-CoV-2 RNA-Carrying Lentiviral Particle Titer Determination and the Feasibility of LFIA for Sensing Lentiviral Particles

**Figure 2:**
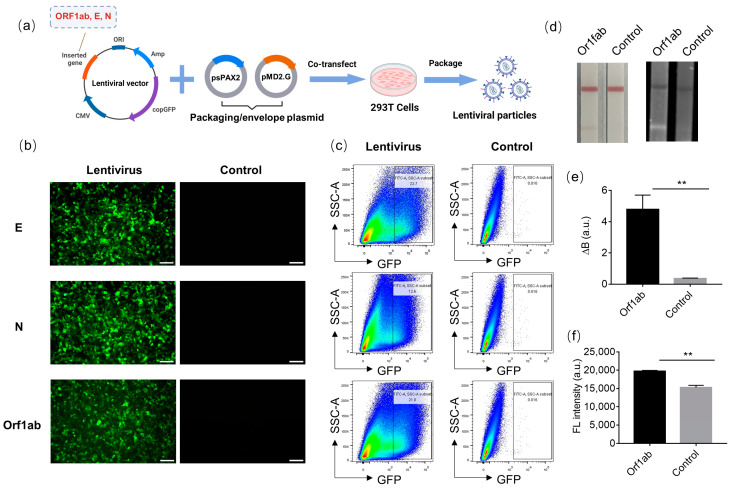
LFIA-based detection of SARS-CoV-2 lentiviral particles. (**a**) A schematic of lentiviral particle packaging workflow created with BioRender.com. (**b**) Representative fluorescent images showing the HEK293T cells transfected with GFP-expressing lentiviral particles at a 1:40 dilution. Scale bar: 50 μm. (**c**) Representative images of the flow cytometric analysis of the HEK293T cells transfected with the GFP-expressing lentiviral particles at a 1:40 dilution. (**d**) Bright-field (left) and fluorescent (right) images of LFIA for ORF1ab lentiviral particle detection. (**e**) Colorimetric and (**f**) fluorescent analysis of (**d**). ΔB = |B_background_ − B_test line_|. N ≥ 3 per group; ** *p* < 0.01.

In this study, lentiviral particles carrying SARS-CoV-2 RNA fragments were produced as targets to examine the performance of the developed LFIA in SARS-CoV-2 analysis. A three-plasmid co-transfection system was utilized in which the lentivirus vectors containing the genes of interest, the envelope-expressing plasmid of pMD2.G, and the packaging plasmid of psPAX2 were co-infected into HEK293T cells to produce the lentiviral particles (Figure 2a). To determine the lentiviral particle titers, the HEK293T cells were transfected with the GFP-expressing lentiviral particles (Figure 2b). As the transfected cells produced green fluorescence, by calculating the GFP-positive cells with a flow cytometer, the titers of the three lentiviral particles were determined to be 2.98 × 10^6^ TU/mL for the ORF1ab lentiviral particles, 2.78 × 10^6^ TU/mL for the E lentiviral particles, and 2.03 × 10^6^ TU/mL for the N lentiviral particles (Figure 2c). To verify the efficiency of lentiviral particle lysis and the feasibility of the LFIA-based RNA capture and detection, we chose ORF1ab lentiviral particle detection as an example (Figure 2d). We found that both the colorimetric and fluorescent modes of the LFIA exhibited a clear band on the test line in the presence of the ORF1ab lentiviral particle group compared with the control group. The observation was also confirmed by comparing the ΔB values (Figure 2e) and fluorescence intensity (Figure 2f) between the two groups, suggesting the good performance of the developed LFIA in SARS-CoV-2 detection.

### 3.4. Optimization of the LFIA’s Fabrication and Operation Conditions for Sensing Lentiviral RNA

**Figure 3:**
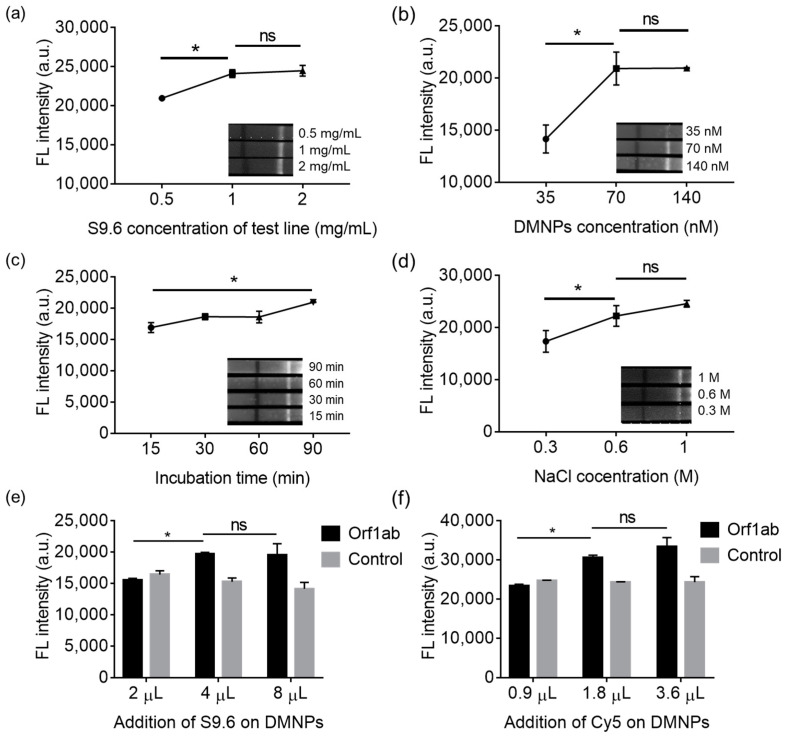
Optimization of the LFIA fabrication and operation conditions for sensing lentiviral RNA. The optimization curves and representative photographs (inset) of (**a**) the S9.6 antibody concentrations (0.5, 1, and 2 mg/mL) on test line, (**b**) DMNP concentrations (35, 70, and 140 nM), (**c**) incubation times (15, 30, 60, and 90 min) of DNA probes with RNA released from SARS-CoV-2 lentiviral particles, (**d**) NaCl concentrations (0.3, 0.6, and 1 M) of hybridization, and the addition of (**e**) S9.6 antibody (2, 4, and 8 μL) and (**f**) Cy5 (0.9, 1.8, and 3.6 μL) onto the GNPs. The ORF1ab lentiviral particles were used in the optimization experiments. N ≥ 2 per group; ns: not significant; * *p* < 0.05.

To achieve a better detection performance of the LFIA, the detection conditions were optimized. First, the concentration of the S9.6 antibody on the test line was optimized. Three concentrations of the S9.6 antibody (0.5 mg/mL, 1 mg/mL, and 2 mg/mL) were dispensed on the test line followed by the lentiviral particle ORF1ab RNA detection. The results show that the fluorescence intensity of the test line increased with the increase in the S9.6 antibody concentration and reached a plateau at 1 mg/mL (Figure 3a), suggesting that increasing the concentration of S9.6 can capture more DNA–RNA hybrids on the test line and the fluorescence signals become saturated when the S9.6 antibody concentration is above 1 mg/mL. Therefore, 1 mg/mL of the S9.6 antibody was thought to be the optimal concentration for the LFIA. Moreover, the concentration of DMNPs utilized was also important for the sensitivity of the LFIA. It was seen that the fluorescence intensity of the test line increased when the DMNPs’ concentration increased from 35 nM and reached a plateau and leveled off when it increased from 70 nM to 140 nM (Figure 3b). The concentration of 70 nM was chosen as the optimal DMNPs concentration. Next, the incubation time of the DNA probe with lentiviral RNA was investigated. With the time increased from 15, 30, and 60 to 90 min, the fluorescence intensity of the test line increased, although there was no difference between the incubation times under 60 min (Figure 3c). Thus, the incubation time of 90 min was chosen as the optimal incubation time for the LFIA. Similarly, the concentration of NaCl was thought to be an effective factor in optimizing the nucleic acid hybridization condition [39]. As shown in Figure 3d, we also found that the more salt added, the more the obvious fluorescent band obtained. To obtain enough DNA–RNA hybrids captured on the test line, the NaCl concentration of 1 M was finally chosen. 

With the above optimal GNP size, the amounts of the S9.6 antibody and Cy5 conjugated on the DMNPs were further optimized, which is important for SARS-CoV-2 detection. First, we altered the volume of the S9.6 antibody from 2 to 4 μL during DMNPs fabrication at a fixed Cy5 concentration of 3.5 mM (Figure 3e). We found that the fluorescence intensity of the test line increased in the detection of the Orf1ab lentiviral particles (10^5^ TU/mL) with the increase in the S9.6 antibody volume. However, the fluorescence intensity of the test line remained unchanged when the volume was above 4 μL. These results suggest that 4 μL of the S9.6 antibody was sufficient for the formed DMNPs to bind with the DNA–RNA hybrids. It is worth mentioning that an excess amount of BSA was also added to the GNPs to improve the dispersibility of the DMNPs and to prevent fluorescence quenching caused by aggregation. To optimize the amount of Cy5 on the DMNPs, we adjusted the volume of Cy5 (0.9, 1.8, and 3.6 μL) and used a fixed S9.6 antibody concentration (Figure 3f). By measuring the fluorescence intensity of the test line after the addition of the Orf1ab lentiviral particles, we found that the fluorescence signal increased when the volume increased from 0.9 to 1.8 μL and then reached a plateau when the concentration was above 1.8 μL, while the background signals remained the same. Therefore, 1.8 μL of Cy5 was determined as the optimal Cy5 volume. The optimized experimental conditions were ultimately used for the following lentiviral RNA detection experiments.

### 3.5. The Sensitivity and Selectivity of LFIA for SARS-CoV-2 Detection

**Figure 4:**
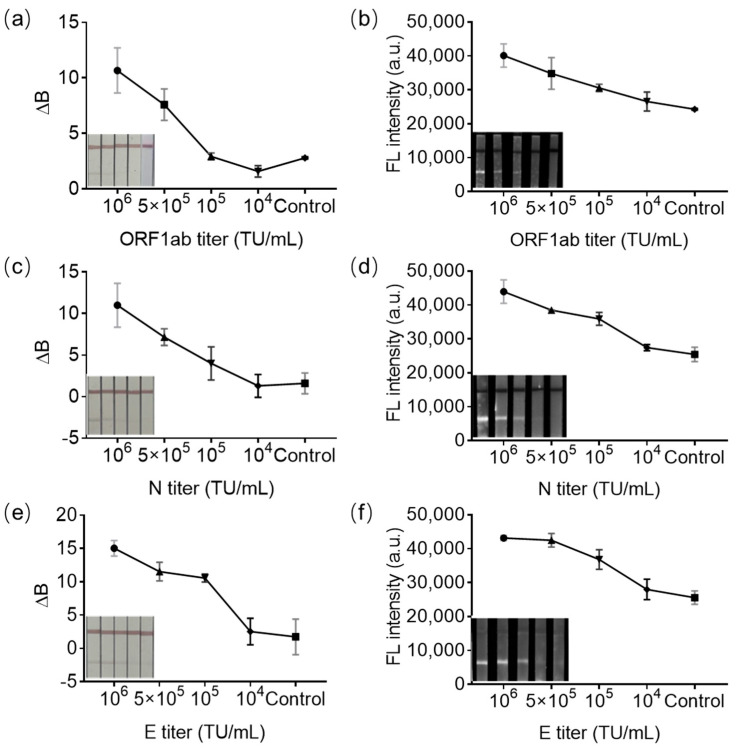
The sensitivity of LFIA in detecting three different types of SARS-CoV-2 RNA-carrying lentiviral particles. The colorimetric analysis and representative photographs (inset) of LFIA for the detection of (**a**) ORF1ab, (**c**) N, and (**e**) E regions of the SARS-CoV-2 RNA-carrying lentiviral particles. Fluorescence analysis and representative photographs (inset) of (**b**) ORF1ab, (**d**) N, (**f**) E regions of the SARS-CoV-2 RNA-carrying lentiviral particles. ΔB = |B_background_ − B_test line_|. N ≥ 3 per group.

Under the optimized conditions, the sensitivity and selectivity of the LFIA for the detection of SARS-CoV-2 RNA were explored. First, the test lines of the LFIA with the addition of three different concentrations of lentiviral particles (10^4^–10^6^ TU/mL) were recorded in the colorimetric mode and the fluorescent mode. For the ORF1ab lentiviral particles, it was found that the value of ΔB and the fluorescence intensity increased with the increase in the ORF1ab titers in the range of 10^4^ and 10^6^ TU/mL and then reached the control level below 10^4^ TU/mL of the ORF1ab titers (Figure 4a,b). The detection trends for the E (Figure 4c,d) and N (Figure 4e,f) lentiviral particles were similar to that of the ORF1ab lentiviral particles in both modes. Thus, the linear range of the assay in dual mode was between 10^4^ and 10^6^ TU/mL for all three lentiviral particles (R^2^ > 0.9). Based on the 3σ rules, the LOD values of the colorimetric analysis were determined as 1.08 × 10^5^, 1.42 × 10^5^, and 1.31 × 10^5^ TU/mL for the ORF1ab, N, and E lentiviral particles, respectively, while the LOD values of the fluorescence analysis were 0.76 × 10^4^, 2.58 × 10^4^, and 1.83 × 10^4^ TU/mL for the ORF1ab, N, and E lentiviral particles, respectively. The fluorescent mode was more sensitive (up to 10 times higher) than the colorimetric mode, suggesting the dual-mode detection capability of the LFIA. 

It is worth mentioning that our developed DMNP-based LFIA platform for SARS-CoV-2 detection has advantages in specific applications, although it is less sensitive than the LFIA based on europium chelate-based fluorescent nanoparticles in Wang’s work [16]. The sacrifice in sensitivity leads to valuable dual-mode detection, which is very important in applications under certain conditions. For example, some research showed that the secondary attack rate of SARS-CoV-2 infection was 17% on average when the viral load was higher than 1 × 10^6^ copies/mL [40]. Usually, the physical titer (copies/mL) was higher than the functional titer (TU/mL) by a factor of 10 to 100 times. Based on this consideration, the LOD of 10^4^ TU/mL in this work was calculated to be 10^5^–10^6^ copies/mL, which is applicable to the situation of high infectivity of SARS-CoV-2 (>1 × 10^6^ copies/mL). Therefore, the developed LFIA could be used in periods of high infectivity to provide important guidance for the home quarantine of COVID-19 patients, thus preventing the spread of infection. In the future, the sensitivity of the fluorescence detection mode could be improved by adjusting the distance between the GNPs and Cy5 [38] or replacing the GNPs with gold nanorods [41].

**Figure 5:**
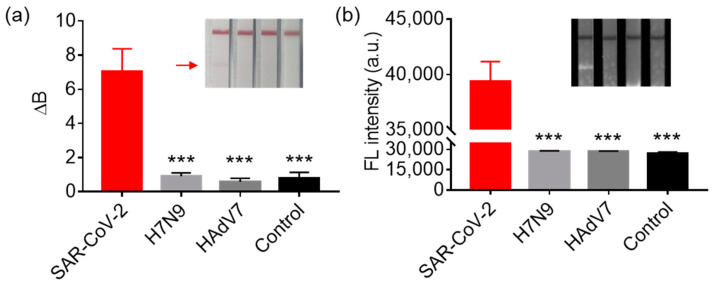
Specificity of LFIA in detecting SARS-CoV-2 RNA-carrying lentiviral particles in comparison with two respiratory DNA/RNA viruses (negative control viruses). (**a**) Colorimetric and (**b**) fluorescence analysis. Inset: Representative photographs with the test line labelled with red arrow. The concentration of H7N9 and HAdV7 viruses was 10^5^ TU/mL. The SARS-CoV-2 group contained 10^5^ TU/mL of ORF1ab-, E-, and N-carrying lentiviral particles to simulate the complete viral genome. The control group refers to the detection buffer. ΔB = |B_background_ − B_test line_|. N ≥ 3 per group; *** *p* < 0.001.

To evaluate whether the LFIA has good specificity for detecting SARS-CoV-2, the adenovirus (HAdV7) and influenza A (H7N9) respiratory viruses were chosen as negative controls. Similarly, these control viruses were first treated with Triton X-100 to release RNA. Furthermore, all three lentiviral particles carrying SARS-CoV-2 RNA fragments of ORF1ab, E, and N were applied to simulate the complete RNA genome of SARS-CoV-2, and the three corresponding probes were added. The results show that only the LFIA with the addition of SARS-CoV-2 RNA-carrying lentiviral particles exhibited an obvious band in both the colorimetric and fluorescence detection modes. On the other hand, the LFIA with the addition of the control viruses of HAdV7 and H7N9 did not exhibit any visible band for any colorimetric or fluorescent signals (Figure 5). The quantitative analysis of the colorimetric and fluorescent bands showed consistent results, suggesting that the LFIA possesses good specificity in sensing the characteristic RNA of SARS-CoV-2 due to the combined use of S9.6 antibodies. 

### 3.6. The Performance of LFIA in SARS-CoV-2 Detection in Saliva Samples

**Figure 6:**
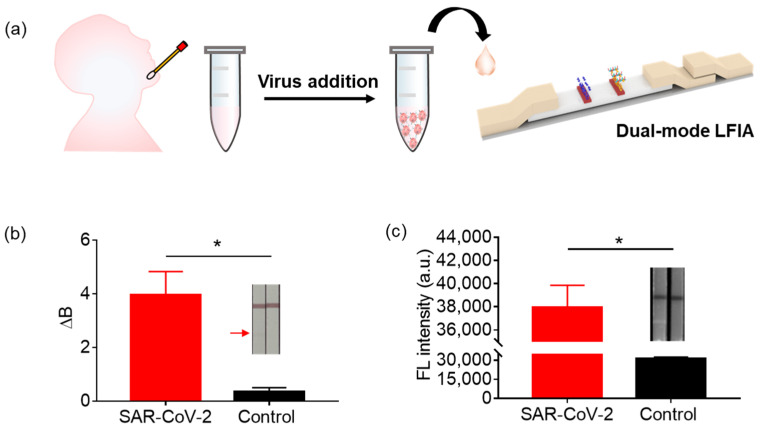
The performance of LFIA in SARS-CoV-2 detection in saliva samples. (**a**) The schematic diagram of the workflow for SARS-CoV-2 lentiviral particles (10^5^ TU/mL of mixture of ORF1ab, N, and E lentiviral particles) spiked in the saliva. (**b**) Colorimetric and (**c**) fluorescence analysis. Inset: Representative photographs with the test line labelled with a red arrow. ΔB = |B_background_ − B_test line_|. N ≥ 3 per group; * *p* < 0.05.

In order to verify the capability of the LFIA for SARS-CoV-2 detection in complex samples, human saliva spiked with 10^5^ TU/mL of SARS-CoV-2 RNA-carrying lentiviral particles was analyzed (Figure 6a). We found that the ΔB of the SARS-CoV-2 group was significantly higher than that of the control group in the colorimetric analysis, although the red band corresponding to SAR-CoV-2 was less obvious to the naked eye compared with that in Figure 5a (Figure 6b). This could be due to the presence of RNase in the complex saliva samples, leading to the degradation of the SARS-CoV-2 RNA [42]. In contrast, the LFIA exhibited a clear band in the fluorescent mode, and the quantitative analysis results also showed significant changes (Figure 6c). These results suggest that the developed LFIA was capable of analyzing the SAR-CoV-2 RNA in the saliva samples, making it a promising point-of-care tool for viral RNA detection.

## 4. Conclusions

In this work, a simple, amplification-free, GNP-based dual-mode LFIA for the direct detection of SARS-CoV-2 RNA was developed. The viral RNA was detected by the designed DNA probes to specifically hybridize with the characteristic RNA regions (ORF1ab, E, and N) of SARS-CoV-2 to form DNA–RNA hybrids. These hybrids were then recognized by the DMNPs to produce colorimetric and fluorescence readout signals. The GNPs with a diameter of 5 nm were confirmed to have the optimal size to produce sensitive dual-mode signals as they emitted the highest fluorescence intensity. Under the optimized conditions, the LFIA presented a linear detection range of 10^4^–10^6^ TU/mL with LOD values of 0.76, 1.83, and 2.58 × 10^4^ TU/mL for the lentiviral particles carrying the ORF1ab, E, and N motifs, respectively, in the fluorescent mode, which was up to 10 times more sensitive than the colorimetric mode. In addition, the LFIA exhibited a good specificity in distinguishing the SARS-CoV-2 lentiviral particles from other respiratory viruses. More importantly, the LFIA was capable of analyzing the SAR-CoV-2 lentiviral particles in saliva. This work demonstrated a simple and easy-to-use LFIA method integrated with dual-mode nanoparticles for sensitive SARS-CoV-2 RNA detection in complexed biological samples. The developed LFIA represents a highly efficient platform for self-testing or clinical detection of SARS-CoV-2 as well as other RNA viruses by further improvement.

## Figures and Tables

**Table 1:** The sequences of the synthesized DNA probes.

DNA Probe	Sequence (5′-3′)
ORF1ab probe	“AACGATTGTGCATCAGCTGACTGAAGCATGGGTTCGCGGAGTTGATCACAACTACAGCCATAACCTTTCCACATACCGCAGACGGTACAGACTGTGTTTTTAAGTGTAAAACCCACAG”
E probe	“CGAAGCGCAGTAAGGATGGCTAGTGTAACTAGCAAGAATACCACGAAAGCAAGAAAAAGAAGTACGCTATTAACTATTAACGTACCTGT”
N probe	“CAGACATTTTGCTCTCAAGCTGGTTCAATCTGTCAAGCAGCAGCAAAGCAAGAGCAGCATCACCGCCATTGCCAGCCATTCTAGCAGGAGAAGTTCCCC”

**Table 2:** The target sequences in the lentiviral plasmids.

Expression Plasmid	Sequence (5′-3′)
pCDH-ORF1ab	“GCAACAGTTCAAGAAATTCAACTCCAGGCAGCAGTAGGGGAACTTCTCCTGCTAGAATGGCTGGCAATGGCGGTGATGCTGCTCTTGCTTTGCTGCTGCTTGACAGATTGAACCAGCTTGAGAGCAAAATGTCTGGTAAAGGCCAACAACAACAAGGCCAAACTGTCACTAAGA”
pCDH-E154	“CGAACTTATGTACTCATTCGTTTCGGAAGAGACAGGTACGTTAATAGTTAATAGCGTACTTCTTTTTCTTGCTTTCGTGGTATTCTTGCTAGTTACACTAGCCATCCTTACTGCGCTTCGATTGTGTGCGTACTGCTGCAATATTGTTAACGTG”
pCDH-N283	“AATTACAAAAATTCAAAATTTTCGGGTTTATTACAGGGACAGCAGAGATCCAGTTTATCGATGCAACAGTTCAAGAAATTCAACTCCAGGCAGCAGTAGGGGAACTTCTCCTGCTAGAATGGCTGGCAATGGCGGTGATGCTGCTCTTGCTTTGCTGCTGCTTGACAGATTGAACCAGCTTGAGAGCAAAATGTCTGGTAAAGGCCAACAACAACAAGGCCAAACTGTCACTAAGAATCGATACTAGTAAGGATCTGCGATCGCTCCGGTGCCCGTCAGTGGG”

## Data Availability

Data are contained within the article.
